# Understanding the Impact of Pain Among Latinx Older Adults

**DOI:** 10.1093/geroni/igaa057.1103

**Published:** 2020-12-16

**Authors:** Calia Morais, Michael Robinson, Roger Fillingim, Emily Bartley

**Affiliations:** 1 University of Florida, Pain Research and Intervention Center of Excellence, Gainesville, Florida, United States; 2 University of Florida, Center for Pain Researach and Behavioral Sciences, Gainesville, Florida, United States; 3 University of Florida, Pain Research and Intervention Center of Excellence, Gainesville, United States

## Abstract

Pain is the number one reason for seeking medical attention, and a top contributor to healthcare costs in the United States. Considerable evidence highlights racial/ethnic disparities in pain with Latinx communities being disproportionally affected at higher rates. Compared to other ethnic minority groups, Latinx older adults are more socioeconomically disadvantaged and experience higher rates of disability and frailty (Garcia, Downer, Crowe, & Markides, 2017). Given the rapidly growing population of Latinx, it is anticipated that the incidence of pain and its associated impact will adversely affect the quality of life of many older adults. The public health focus on improving pain management with non-opioid treatment options also highlights the need to provide culturally responsive care for Latinx older adults. However, limited efforts have addressed this important target for intervention. Following the biopsychosocial model of pain, this presentation will provide an overview of biological, psychosocial and cultural factors influencing pain disparities among older adults. Findings from experimental and clinical settings will be discussed. The presenter will also review current evidence highlighting the preference for self-management strategies for pain management among Latinxs, as well as the need to increase accessibility to psychosocial treatments for chronic pain, such as cognitive behavioral therapy for pain (CBT-pain). Furthermore, preliminary findings from a qualitative study will be presented to illustrate the need to offer culturally adapted treatments for pain management (Torres, Thorn, Kapoor, & DeMonte, 2017). Future research is needed to study sociocultural factors influencing pain disparities to help identify modifiable targets for intervention.

